# The Profession's Friends—The S. S. White Co., and the International Tooth Crown Co.

**Published:** 1900-02

**Authors:** 


### Editorial, Etc.

### THE PROFESSION'S FRIENDS—THE S. S. WHITE CO., AND THE INTERNATIONAL TOOH CROWN CO.

Our attention has beeen called to a recent circular sent out by the S. S. White Co., which professes to be an interpretation of the decision in the recent case of the Crown Co., vs. Jas. Orr Kyle, the defendant in the Crown Co.'s last suit. This circular is unfair, tricky and misleading. It is intended to convey the impression that the dentists stand in no danger of litigation from the International Tooth Crown Co., except upon the Low bridge patent, which expired in March, 1898, and that they are in very little danger from even that patent.

Nothing is further from the truth. The Crown Co., have at least thirty-eight patents, for the most part unexpired, relating to crown and bridge-work and other artificial dentures. The settlements that have been made thus far by the Crown Co., with certain members of the Protective Association, and the release and license given, relate to fifteen patents only. This bears out our previous statement and confirms the charge of misrepresentation against the S. S. White Co., in their circular.

From a reading of said circular it would appear that its sole object and intention is to discourage the non-members from uniting with us in the Association, and to intimate to the members that they have been frightened into joining when no danger existed of their being sued.

Every dentist knows that he cannot be held for work infringing on a patient after it has expired. This has been frequently stated by us in public meetings and circulars, and we have also stated the length of time for which the profession are liable, in case the patient should prove valid.

It has been impossible for us to make any distinction between dentists who were old in the manufacture of bridge-work and the younger men who had used the method but a short time before the patent on it expired. The longer time and more work a dentist did the larger amount the Crown Co., could collect from him, but a man who infringed a patent one day before its expiration could be held for one year's license and royalty.

The S. S. White Co., circular intimates that the whole matter is too trifling for the dentists to take any interest in. We would merely point to the twelve year's litigation which the Protective Association has had with the Crown Co., and with several other patent organizations, and the fact that although this patent has expired for nearly two years the Crown Co., are far more active in the bringing of suits against the dentists for past royalty and license fees than they were before the patent's expiration. No one knows better than the officers of S. S. White Co., and their attorneys that no more harrassing form of litigation can be put upon the dental profession than the numerous suits which are now being brought in various states. The pretended fear of the S. S. White Co., that they or some of their patrons would be sued by the Crown Co., because of the manufacture and use of "the Logan and other porcelain crowns, and the gold cap crowns" sold by them, is not only insincere but ridiculous.

The action of the Tooth Crown Co., in attempting to intimidate members, and the cause taken by their apparent allies, the S. S. White Co., have been so contemptible and misleading that we feel strongly tempted to open wide the doors aud give every dentist in the profession, who has not already done so, a chance to join the Protective Association. The books were closed at the solicitation of the membership, but several prominent members and societies have suggested that to reopen the doors might perhaps after all be the wisest course. Our inclination has always been not to limit protection, but the members objected, and rightly, to paying for the defense of those who contributed nothing.

The action of the S. S. White Co., in this instance is in strict accordance with their past methods. The Trust sees the menace to its interests in having the profession banded together, and while they dare not come out so openly, and through their traveling salesmen misrepresent and belittle the Protective Association as they have done in the past, they adopt a more subtle and contemptible course of warfare against our organization. If the S. S. White Co., think the Protective Association is on the right track, why do they not come out and say so? Or if they really believe the malicious statements which they have circulated about us, why have they not the courage of their convictions to openly denounce this organization?

We shall shortly carry the war into the enemy's camp, and issue a detailed communication, showing in small part the vast sums of money which the S. S. White Dental Mfg. Co., has mulcted from the profession in the shape of royalties upon worthless patents, and furthermore, expose the various subterfuges and ledgerdemain which that company makes use of to conceal the fact that the money is so taken from the dentists. Unlike our traducers, we have facts upon which to base our accusations and not draw upon imagination.—*Dental Digest.*

